# Mechanical Compression Effects on the Secretion of vWF and IL-8 by Cultured Human Vein Endothelium

**DOI:** 10.1371/journal.pone.0169752

**Published:** 2017-01-12

**Authors:** Dar Weiss, Sharon Avraham, Ruth Guttlieb, Lee Gasner, Alina Lotman, Oren M. Rotman, Shmuel Einav

**Affiliations:** 1 Department of Biomedical Engineering, Faculty of Engineering, Tel Aviv University, Tel Aviv, Israel; 2 Department of Biomedical Engineering, Stony Brook University, Stony Brook, New York, United States of America; Centro Cardiologico Monzino, ITALY

## Abstract

Short peripheral catheters are ubiquitous in today's healthcare environment enabling effective delivery of fluids and medications directly into a patient's vasculature. However, complications related to their use, such as short peripheral catheter thrombophlebitis (SPCT), affect up to 80% of hospitalized patients. While indwelling within the vein, the catheters exert prolonged constant pressure upon the endothelium which can trigger inflammation processes. We have developed and studied an in-vitro model of cultured endothelial cells subjected to mechanical compression of modular self-designed weights, and explored their inflammatory response by quantification of two key biomarkers- vWF and IL-8. Evaluation was performed by ELISA immunoassay and processing of vWF-labeled immunofluorescence images. We found that application of weights correspond to 272 Pa yielded increased release of vWF and IL-8 up to 150% and 250% respectively, comparing to the exertion of 136 Pa. Analyses of the immunofluorescence images revealed significantly longer and more extracellular vWF-strings as well as higher intensity stained-pixels in cells exposed to elevated pressures. The release of both factors found to be significantly dependent on the extent of the exerted pressure. The research shed a light on the relationship between induced mechanical compression and the pathogenesis of SPCT. Minimizing, let alone eliminating the contact between the catheter and the vein wall will mitigate the pressure acting on the endothelium, thereby reducing the secretion of inflammatory factors and lessen the incidence of SPCT.

## Introduction

Intravenous (IV) procedure involves an invasive insertion of a short peripheral catheter (SPC) into a peripherally located vein in the upper hand or forearm [1, 2]. During the SPC indwelling within the vein, it constantly contacts and exerts local mechanical pressure (compression forces) upon the endothelial cells (ECs) layer lining the inner vein wall [3, 4]. The main complication associated with these catheters is short peripheral catheter thrombophlebitis (SPCT) which can occur in up to 80% of hospitalized patients receiving IV therapy [5]. Thrombophlebitis is a generic term for a sterile inflammation of the vein wall usually characterized by edema, erythema and thrombus formation. To date, the pathogenesis of SPCT is unknown. We believe that the biomechanical interaction between the SPC and the vein wall promotes inflammatory response of the ECs. The SPC irritates and activates the ECs, which in response trigger a complex network of mediators and factors to initiate inflammatory processes. Among these factors and of those significant, are the pro-inflammatory chemokine interleukin-8 (IL-8) and von Willebrand factor (vWF), a multimeric glycoprotein which is routinely packaged and stored in specialized exclusive organelles termed Weibel-Palade bodies (WPB) within the ECs. These highly-organized vesicles function as a "first- aid emergency kit" in cases of injury or mechanical trauma and secreted through exocytosis in response to cell-external agonist stimulation. When released *in-vivo*, the folded vWF multimers are rapidly anchored to the surface of the ECs as ultra large hyperactive strings [6]. The role of these strings in pathophysiologic processes has been extensively studied [7–9]. It is believed that these strings contribute to inflammatory diseases such as thrombotic thrombocytopenic purpura (TTP) [9], arterial thrombosis [10], deep vein thrombosis [8], and have prothrombotic effect by promoting leukocyte and platelets adhesion and rolling on the ECs surface [7, 11].

The production of IL-8 has been shown to have several consequences on the tissular environment. IL-8 has, *in-vitro*, an attractant activity for neutrophils, lymphocytes, basophiles, and eosinophils [12, 13]. Moreover, IL-8 induces activation and trans-endothelial migration of neutrophils [14, 15]. It may also trigger the activation of basophils involved in the pathogenesis of the late inflammatory reaction [16].

Recently, we laid the groundwork, both *in-silico* [3] and *in-vitro* [4], for the contribution of the biomechanical interaction between the SPC and the endothelial layer to the evolution of SPCT. Here we expand our experimental *in-vitro* model, and investigate the sensitivity of cultured ECs to gradually-increased mechanical compression load in enhanced comprehensive biological experiments. To the best of our knowledge, no information exists as to whether externally-applied mechanical pressure induces inflammation processes by modulating the local release of vWF and IL-8 from vein endothelium. The present study was designed to investigate whether, and how the stimulation of cultured vein endothelial cells by externally-applied mechanical pressure modulates the release of these aforementioned key inflammatory biomarkers, thereby shedding light on the relationship between SPC-induced mechanical compression and the pathogenesis of SPCT.

## Materials and Methods

The inflammatory response of ECs to mechanical compression stimulation was investigated. For this purpose, the secretion of two key inflammatory biomarkers, vWF and IL-8, following exposure to various weights and increasing incubation times, was quantified. Evaluation was performed by ELISA immunoassay and analysis of vWF-stained immunofluorescence images.

### EC culture

Human Umbilical Vein Endothelial Cells (HUVEC; PromoCell) were grown in endothelial cell growth medium (PromoCell) supplemented with 100 U/ml Penicillin, 0.1 mg/ml Streptomycin and 12.5 U/ml Nystatin (Biological Industries, Beit Haemek, Israel), and maintained at 37°C in a 5% CO2 humidified incubator. For mechanical compression experiments, HUVEC were seeded onto tissue cultured 12-wells plates at density of 6x10^4^ per well and used 2 days later, after attaining confluence (~90%). HUVEC were routinely used between the fourth and seventh passage.

### Woven wire mesh weights

**Fig 1:**
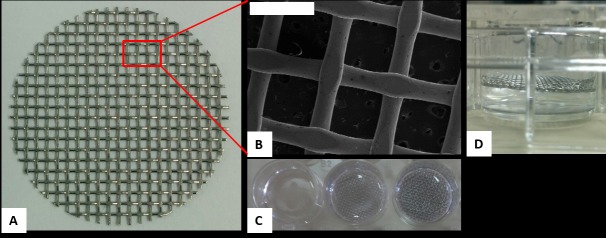
A. Woven-wired disk shaped mesh weight. B. Scanning electron microscopy image of the mesh. Each Contact point is ellipses shape with an area of ~0.5 mm^2^. Scale bar length is 1mm C. The mesh completely fit each well in a 12-wells culture dish (Top view). The rightmost well contains 4 meshes and the middle one 2 meshes, corresponding to approximately 272 Pa and 136 Pa, respectively. The naive control group is mesh-free (left well). D. Side view of a culture plate, demonstrating the position of the mesh during cyto-toxicity tests (no contact with the ECs monolayer)**.**

For mechanical pressure exertion, disk shaped weights in the form of woven wire mesh were designed (Fig 1A). The meshes are composed of biocompatible stainless-steel 316 (0.3 mm wire diameter) with 50% porosity. Each mesh is 0.4mm in thickness, and has an effective weight (within the growth medium) of ~ 3.5 mN (S1 File), and diameter of 21.9mm, so it is completely fit each well in the 12-wells culture plate (Fig 1C). The effective contact surface of the mesh with the bottom of the culture plate was ~50 mm^2^ (S1 File, S2 Fig), as was estimated by scanning electron microscopy (Fig 1B). Prior to the experiments all weights were sonicated in a designated ultrasonic bath for 30 minutes, and then sterilized in an autoclave for two and a half hours. The following treatment groups were assembled for all the experiments reported herein: control (weight-free), 2 units of mesh and 4 units of mesh corresponding to ~136 Pa and ~272 Pa, respectively.

### Meshes toxicity test

Cyto-toxicity effect of the woven-wire meshes toward the HUVEC, as well as Apoptosis/Necrosis processes (and disruption of the cell monolayer) as a consequence of the mechanical compression exerted by the mesh weights, were both performed using Annexin V- Propidium Iodide (PI) kit (Tali^™^ Apoptosis Kit), followed by a measurement of cells fluorescence by flow cytometry analysis (For toxicity examination we further used Alamar Blue^™^ viability test-see S2 File). HUVEC, passage 7, were plated in 12-wells plates, at 60,000 cells per well in a culture medium and grown for 3 days, reaching confluence (~90%). Then, zero (control) to four woven-wire meshes were placed on top of the cells (for toxicity test, the meshes were held inside each well without contacting the cell monolayer- Fig 1D) for 0–96 hours in 37°C. Cells pretreated with 100 μL of hydrogen peroxide (H_2_O_2_) for 10 minutes and unexposed to any mesh weights, constituted another treatment group (negative control). Following predetermined time points (24,48,72,96 hours), the meshes and the medium were removed, the cells were washed using PBS, trypsinized and marked with Annexin and PI according to the kit protocol. Samples were analyzed using a BD^™^ LSR digital flow cytometer. The Flowing software (Turko University, Finland) was used for data analysis.

### vWF and IL-8 secretion in response to mechanical stress stimuli

The secretion of vWF and IL-8 in response to induced mechanical pressure was quantified in a separate independent experiment for each factor. For both experiments, HUVEC were plated in 12-wells plates and grown to confluence as described above. On the experiment day, the growth medium was aspirated; each well was washed twice with 1 ml of phosphate-buffered saline (PBS), and 2 ml of fresh growth medium were added. ECs were then incubated at 37°C in 5% CO_2_ in the presence of the following treatments (Fig 1C): 2 meshes, 4 meshes, naive control (no meshes) and histamine (100 μM)—a known endothelial-cell WPB secretagogue [17–19], as a positive control. The number of meshes was varied in order to expose the cells to elevated levels of mechanical pressure. For the vWF experiments, ECs were incubated for 30, 60, 120 and 180 minutes (one culture plate for each incubation time point) while for the IL-8 experiments incubation times were 24, 48, 72 and 96 hours. Immediately at the end of each incubation time, the supernatant of each treatment was collected and stored at -20°C until quantification analysis.

vWF and IL-8 secretion levels in the medium were determined each with a designated commercially available ELISA reagent set using a microplate spectrophotometer reader (Multiskan^™^ GO, Thermo) at absorbance of 450 nm and 492 nm for the vWF and IL-8, respectively, according to the manufacturer’s instructions (vWF ELISA set- Biotest; IL-8 ELISA set-PeproTech).

### Immunofluorescence stains (vWF strings stain)

For immunofluorescence studies HUVEC were grown on 0.1% Gelatin (Sigma-Aldrich^®^) pre-coated glass coverslips. Cells were exposed to 0, 2 and 4 meshes for duration of 2, 5 and 10 minutes; histamine (100 μM) was used as a positive control.

At the end of each incubation time, medium was aspirated and cells were rinsed with PBS (Cells were not permeabilized). For vWF staining, the cells were marked with sheep anti-vWF antibody conjugated to fluorescein isothiocyanate (FITC; ab8822, Abcam^®^), 1:200 diluted in PBS, for 30 min incubation at 37°C. Cells were then washed with PBS and fixed in 3.7% paraformaldehyde solution for 15 min at room temperature. Coverslips were placed on glass slides containing DAPI mounting medium (Santa Cruz Biotechnology, Inc.) in order to visualize cells' nuclei. Slides were stored at 4°C in a sealed box until microscope examination. Images of extracellular- anchored vWF and cells' nuclei were sequentially acquired with a LSM510 META confocal microscope (Zeiss) through either a X25 (NA 0.7) or X63 (NA 1.4) oil-immersion objective lens with excitation and emission wavelengths of 488nm and 525nm for the FITC (green channel) and 405nm and 420nm for the DAPI (blue channel), respectively. Each image was acquired with resolution of 1024x1024 pixels and was averaged eight times in order to reduce noise and improve image quality. Five random fields were acquired per each coverslip and per each magnification.

### Image processing analysis

The number and length of individual vWF strings visualized by fluorescence were analyzed using ImageJ (National Institutes of Health) imaging software. The strings lengths were measured following calibration of distance units (pixels to μm) with LSM image browser software (Zeiss).

For quantification of the secreted vWF intensity levels (the green channel) images were digitized to 256 gray levels and analyzed using MATLAB^®^ to compare intensity distribution trends between treatment groups. Next, the amount of secreted vWF-stained pixels and the number of cells' nuclei per each field was counted using a bespoke MATLAB^®^ code in order to calculate normalized amount of vWF per each field.

### Statistical analysis

Secretion levels were expressed as the percentage changes in respect to the naive control at the shortest incubation time. Due to the skewed distribution, values underwent Lan transformation, to obtain an approximate normal distribution. To evaluate the effect of weight (dose) and incubation time on secretion, a two-way ANOVA model was implemented followed by Tukey HSD post hoc test. Interactions were not included in the final model as they were found to be statistically insignificant. For experiments including H_2_O_2_, a three-way ANOVA model was applied. Significance level was defined as *p* = 0.05. A two-way ANOVA model (followed by Tukey HSD post hoc) was also implemented for analysis of the amount of vWF-stained pixels derived from the immunofluorescence images, whereas non parametric Kruskal-Wallis test followed by Dunn post hoc test was used for the analysis of the Intensity levels, vWF strings' length and number. Analyses were carried out using SPSS software, version22.01.

## Results

### Toxicity tests

Cyto-toxicity of the mesh material composition was ruled out as ECs remained intact and viable following the indwelling of mesh weight (for all examined incubation times). Exposure of the cells to the mechanical compression of either two or four units of mesh did not induce marked apoptosis or necrosis events. More than 90% of the cells which were exposed to 4 meshes for 96 hours were both PI and Annexin-V negative, same as obtained for the control (no mesh) and H_2_O_2_ (used as a negative control for the ELISA experiments) groups following 96 hours incubation (detailed FACS and viability test results- S2 File, S3 Fig).

### vWF and IL-8 secretion

A total of 7 experiments were conducted to evaluate vWF secretion in response to 0, 2 or 4 units of mesh weights for incubation times ranging from 30 to 180 min. Secretion was compared to positive (histamine) and negative (H_2_O_2_) controls. Out of 7 experiments, 100% demonstrated a significant increased secretion with increased number of weights (*p*≤0.015) and incubation time (*p*≤0.001). All experiments demonstrated a high coefficient of determination (0.872 ≤R^2^≤ 0.967).

**Fig 2:**
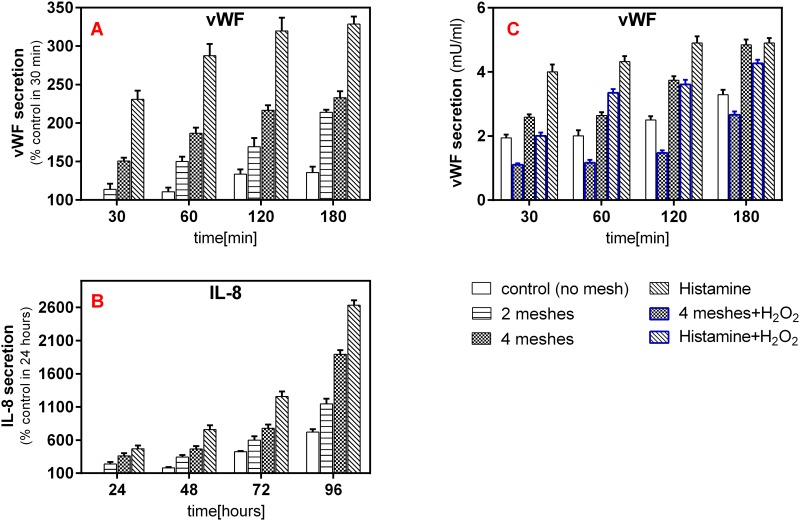
Examples of experimental results. vWF (A) and IL-8 (B) release into the cultured medium from HUVEC exposed to mechanical pressure of 136 Pa (2 meshes), 272 Pa (4 meshes) and histamine (positive control). Data is expressed as the percentage increase versus the control group in the shortest incubation time slot (30 minutes for the vWF and 24 hours for the IL-8) which was each set as 100%. C. An example for vWF experiment involving H_2_O_2_ as a negative control. The 4 meshes and the histamine groups were pretreated with 100 μL H_2_O_2_ for 10 minutes. The secretion, in this case is expressed in units of concentration. 4 experiments demonstrating the effect of H_2_O_2_ were conducted for each of the factors.

Exertion of 4 meshes yielded significantly increased secretion compared to the 2 meshes in 6 out 7 experiments (*p*≤0.05), with values ranging between 117% to 150%. The 2 meshes group induced significantly increased secretion (*p*≤0.001) over the naive group in all experiments, with values ranging between 124% to 213%. Results of one experiment are reported in Fig 2A as the percentage increase in respect to the control group in 30 minutes time point.

For IL-8 secretion, 9 experiments were conducted at incubation times ranging from 24 to 96 hours. All experiments demonstrated significant time and treatment (number of weights) effect (*p*<0.001) with high coefficient of determination (R^2^≥0.895).

Example of one experiment can be seen in Fig 2B. Overall, IL-8 production was significantly higher in cells exposed to 2 meshes as compared to control (*p*<0.032, Tukey post hoc test) and in cells exposed to 4 meshes compared to 2 meshes (*p*<0.032) at all incubation times, in all experiments. The values ranged between 139% to 252% and between 133% to 294% for the comparison of 2 meshes to control and 4 meshes to 2 meshes, respectively.

To verify that the secreted IL-8 and vWF are both originated in the WPB and not derived from a possible unregulated mechanism (cell disruption or lysis) induced by the exposure to the mesh weights, a separate independent series of experiments was carried out to measure their secretion in the presence or absence of 10 minutes pretreatment with 100μL H_2_O_2_ (negative control)—a known inhibitor of WPB exocytosis [20]. It was found that incubation with H_2_O_2_ significantly decreased the secretion of both the vWF (*p*<0.001) and IL-8 (*p*<0.003). Results for one experiment for vWF are shown in Fig 2C. It can be seen, that the secretion of vWF in the 4 meshes H_2_O_2_ group was more than two-fold lower comparing to the 4 meshes group (*P*<0.001), for all incubation times, suggesting that the secretion is associated with enhanced WPB exocytosis pathway, which was induced by the weights stimulation. The histamine treated group showed the highest secretion amount in most of the experiments (both vWF & IL-8), indicative of their validity. In several experiments, the amount of the secreted biomarker was even recorded to be higher in cells treated with 4 units of weight than those with histamine.

### Immunofluorescence studies

**Fig 3:**
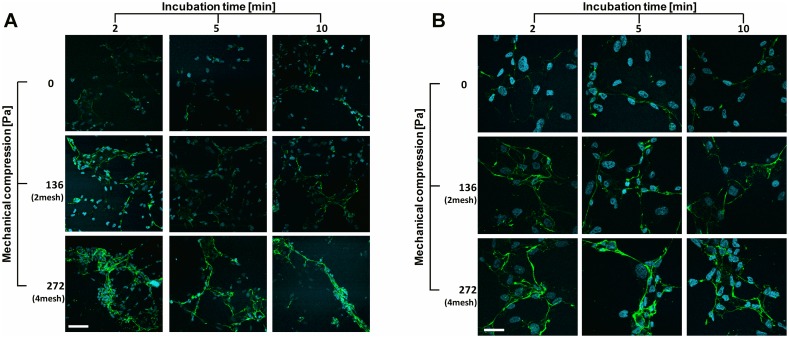
HUVEC were exposed to 0, 2 and 4 meshes for 2, 5 and 10 minutes. Extra-cellular vWF strings were labeled with anti-vWF antibodies (green) and cells' nuclei with DAPI (blue). Representative confocal images of mechanical pressure induced—vWF release from HUVEC for (A). x25 magnification and (B). x63 magnification, were imaged by confocal laser scanning microscopy. Bars represent 50 μm and 20 μm for x25 and x63 magnifications, respectively.

The correlation between vWF release and the induced mechanical compression was also studied using analysis of immunofluorescence images. Total of 839 images were acquired and blindly analyzed to derive three different parameters for each treatment group: normalized (per number of cells) amount of secreted vWF, number and length of anchored-secreted vWF strings, and staining intensity levels of the strings. The results of the immunofluorescence staining are presented in Fig 3.

### Normalized amount of secreted vWF

**Fig 4:**
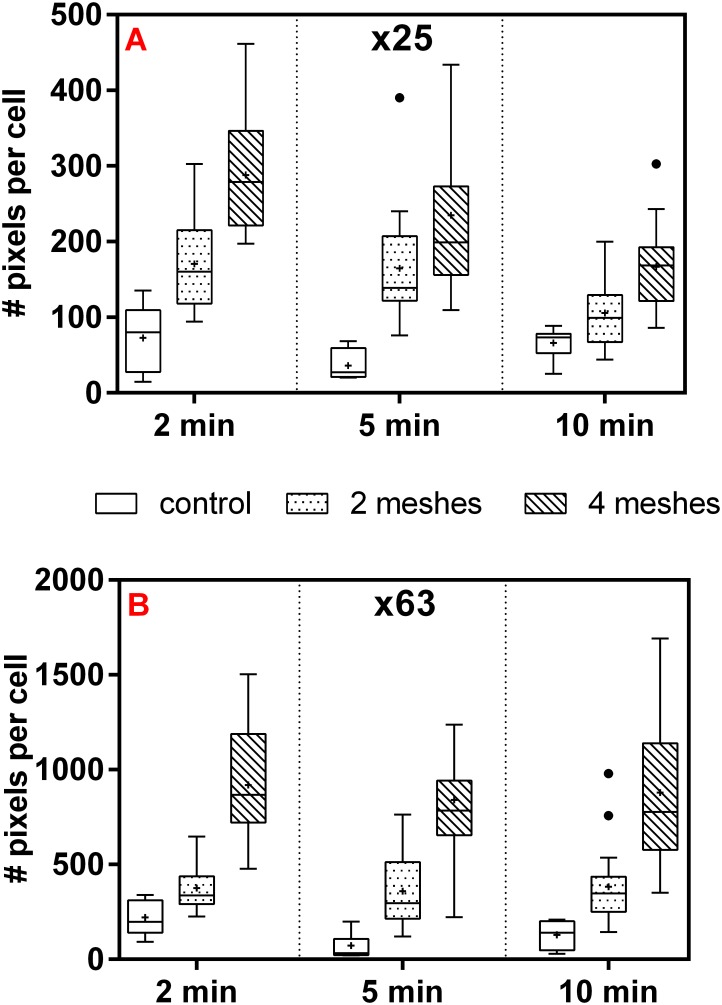
Box plots demonstrating the normalized amount of secreted vWF (Number of pixels per cell) for the x25 magnification (A) and for the x63 magnification (B). HUVEC were exposed to 0, 2 or 4 units of mesh for 2, 5 or 10 minutes followed by incubation with anti-vWF antibody for 30 more minutes. The amount of stained pixels and the number of cells' nuclei were counted per each field to yield normalized secretion for each of the examined treatment group. For each box the "+" sign denotes the average and the median is identified by the line inside the box. The top and bottom of the box are the 25^th^ and 75^th^ percentiles of the samples, respectively. The length of the box represents the inquartile range, outliers values (between 1.5 IQR and 3 IQR from either side of the box) are displayed with filled dots. Bars represent minimum and maximum (Five number summary). Each box plot group (i.e. 2 meshes, 5 minutes incubation time, x63 magnification) represents at least 12 independent experiments and each data sample represents an average of five randomly selected fields within the same cover-slip.

Box plots representation of the normalized amount (pixel count) of the secreted vWF for both magnifications (x25 and x63) and all examined incubation time points are shown in Fig 4. The results clearly demonstrated a significant increase of counted pixels (correspond to secreted amount of vWF) with increasing mechanical compression, for both magnifications. For the x63 magnification (B) for example, the average pixel count for the 4 meshes group, in all incubation times, was 4 to 5 fold higher than the control (*p*<0.001, Tukey post hoc test) and 2–3 folds higher than the 2 meshes group (*p*<0.005). A clear separation between the values was further observed between 2 meshes group and control (*p*<0.003). Significant effect of the exerted pressure was similarly obtained for the x25 magnification (0.815 ≤R^2^≤ 0.897). Time- dependency, on the other hand, was only significant for the x25 magnification. It was further noticed in this regard, that the highest (average) secreted amount for the 2 and 4 meshes groups was obtained after 2 minutes incubation whereas after 10 minutes, it was the lowest (*p*>0.0001 and *p* = 0.044 for 4 and 2 units of mesh, respectively). Control group showed no significant time-dependency.

### Strings length and number

**Fig 5:**
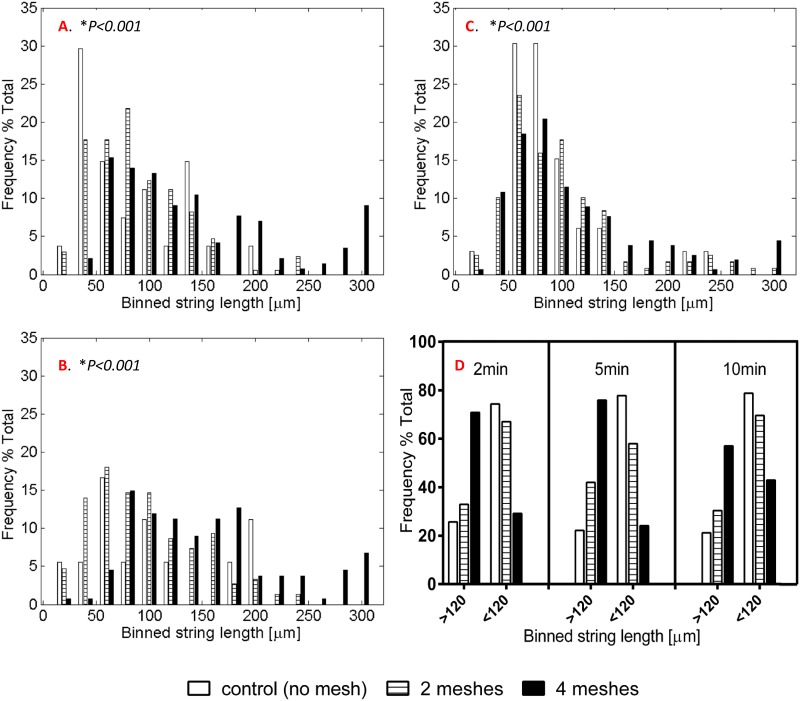
Distribution of the vWF strings length released from cells exposed to 0, 2 or 4 meshes for (A) 2 minutes, (B) 5 minutes and (C) 10 minutes. The strings were observed under x25 enlargement measured using ImageJ software and binned to 20-μm divisions. D. Distribution of strings longer or shorter than 120 μm, for all the examined time points. *p<0.001 by Kruskal-Wallis test.

Length distributions for each examined incubation time at x25 magnification are presented in Fig 5. It was found that for all incubation times the majority of strings produced under 4 mesh exertion were significantly shifted toward the upper end of the length scale while control cells mostly yielded short length strings (Dunn post hoc test, p<0.01). This observation is more clearly shown in Fig 5D where the mean calculated strings length was set as a threshold value (120μm), and the length distribution re-presented accordingly. At 2 and 5 minutes time points, over 70% of the total strings longer than 120 μm belonged to the 4 mesh group, whereas only about 20% were observed in the control. Strings spreading over less than 120 μm mostly appeared in the control group, as approximately 80% of the total at each time point were those of the control. The length calculation analysis was only performed on the x25 magnification images set since some of the strings within the x63 magnification images exceeded the matrix size.

**Fig 6:**
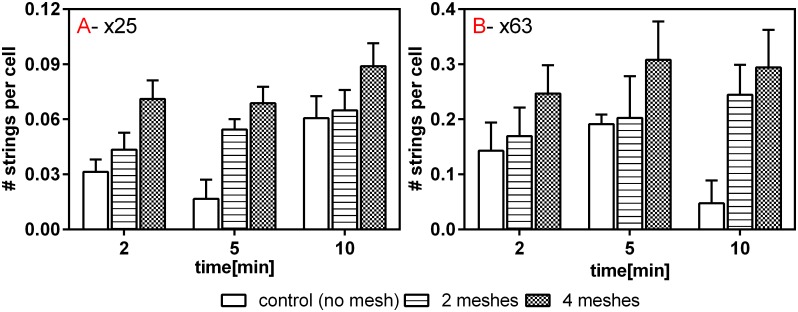
Number of strings normalized in the cells' number per field of view, A. x25 magnification and B. x63 magnification. Data was collected from five fields of view taken from at least 12 separate experiments. Error bars represent Standard deviations.

The number of strings found in each representative field for each of the various treatment groups and incubation time was normalized per the number of cells within the designated field. The results are depicted in Fig 6 for both magnifications. The 4 meshes group induced a significantly greater amount of strings than that of the 2 meshes group for incubation times of 2 and 10 minutes in the x25 magnification and 2 and 5 minutes in the x63 magnification (*p*< 0.01 for all significant comparisons, Dunn post hoc test). The control group exhibited significantly lower amount of strings compared to the 4 meshes, for both magnifications and all incubation times (*p*<0.001). Comparison between the control and 2 meshes groups reached statistically significant difference (*p*< 0.01) with the exception of 5 minutes and 10 minutes incubation times in x63 and x25 magnifications, respectively. Neither the strings length nor their amounts were found to be time dependent.

### Intensity staining analysis

**Fig 7:**
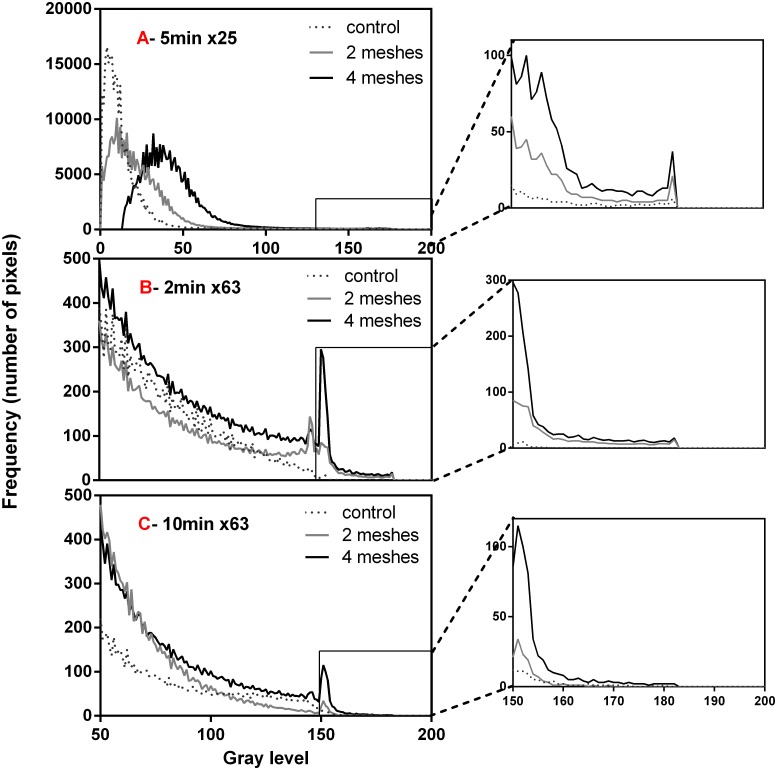
Intensity levels histograms of the tested treatment groups for 3 representative combinations of magnification and incubation time (specified on top of each graph). Each histogram curve represents the average of at least 12 experiments. The x-axis of graph (C) refers also to (B) while the range of gray level between 150 and 200 appears magnified to the right of each graph.

Topographic color maps (S1 Fig) for each mesh group and incubation time were generated and the corresponding averaged histograms (gray level maps) were calculated and are partly presented in Fig 7 (for 3 different combinations of magnification and incubation time). The two bottom histograms (B and C) are shown for gray levels starting at 50 in order to enhance the differences observed in higher levels, where an example for a full-scale histogram is shown in Fig 7A. The results suggest that higher intensity levels pixels (upper end of the gray level axis) were mostly existed in the 4 meshes group followed by the 2 meshes group while only negligible amount was found in the control. For this group (control) all distributions were shifted towards the lower end of the gray levels axis as can be seen in Fig 7A (*p*<0.01 by Kruskal-Wallis test followed by Dunn post hoc, for all comparisons between the weight groups for all magnifications and incubation times). The intensity difference between the treatment groups in the higher gray levels can be seen clearer in the magnified graphs (on the right) which present the gray level domain above 150. Time dependency was not found to be statistically significant.

## Discussion

The present study examined the effect of mechanical compression on the inflammatory response of ECs which was evaluated by the production of vWF and IL-8.

ECs constitute the principal source for vWF and IL-8. They are both stored within distinctive WPB organelles, where vWF is the major constituent [21] and the IL-8 only found in small quantities (it is also localized in the Golgi apparatus). When triggered, the WPB migrate towards the cells surface, fuse with the membrane and "spill" their content through exocytosis. While IL-8 cytokines are secreted to the circulation as a pure pro-inflammatory marker, secreted vWF, which correspond to ultra-large multimers strings, remain anchored to the plasma membrane and play a role in hemostasis as mediators for platelet aggregation which result in the formation of a platelet plug.

The present results indicate that the release of both vWF and IL-8 by ECs is significantly influenced by external mechanical pressure and is dose-dependent (dependent on the level of the exerted pressure). Comparing the relative secretion between the meshes groups of both vWF and IL-8 indicate on significant enhanced secretion with increasing mechanical load. This trend was obtained independently in both methodologies (ELISA and immunofluorescence).

It is worth noting that the obtained absolute values of the secreted biomarkers and the immunofluorescence parameters are not significant as much as the obtained relative trends between the experimental groups which validate our hypothesis.

Evaluation of the HUVEC response to the applied pressure was done using two independent but complementary methodologies: the ELISA method which provided the amount of secreted vWF and IL-8, and advanced analysis of immunofluorescence images which enabled a supplementary quantification of the vWF strings- parameters such as number, length, and intensity of the vWF staining, which cannot be obtained by the ELISA. The amount of vWF-labeled pixels, was also derived through image processing. It should be noted that the amount of vWF quantified by ELISA only referred to the protein released to the supernatant unlike the EC-anchored strings which were quantified in the immunofluorescence analysis. Hence, the amount of released vWF as was assessed by the ELISA and the amount of vWF-stained pixels in the immunofluorescence images are not comparable. Since we were only interested in the extracellular anchored-vWF, we added the immuno-labeled antibodies immediately following the incubation period with the meshes, and prior to fixation. The reverse process (staining following fixation) was found to pierce the cells membrane allowing the antibodies to penetrate the cells and leading to unwanted staining of intracellular vWF located within the WPBs.

The use of two different magnifications (x25 and x63) in the confocal images acquisition process was aimed to ensure the reliability and robustness of the analysis. The x25 magnification was shown the be optimal for the strings length analysis since the corresponding field of view included the full length of the strings, as opposed to the x63 magnification in which, due to field of view limitation, appeared fragmented. On the contrary, the x63 magnification provided better resolution and less noisy images, allowing more reliable analysis in terms of pixel count and intensity evaluation.

In static *in-vitro* conditions, the vWF strings are progressively cleaved by the endogenous (originated in the endoplasmic reticulum and at the Golgi apparatus) enzyme ADAMTS13, which accumulates in the cultured medium[22]. Turner et al. showed that increasing incubation times from 2-to-10 minutes resulted in smaller vWF strings size [23]. Since one of the examined parameter in the current study was strings length, times scales for the immunofluorescence experiments were chosen to be short incubation intervals (scales of minutes) in which the majority of the anchored strings have not been proteolitically processed by the enzyme. For the ELISA purpose, longer incubation times were allowed, especially for the IL-8, as it secreted in scant amounts (hundreds of picograms).

Elevated amounts of both biomarkers with increasing incubation time were observed in the ELISA quantification, whereas in the immunofluorescence analysis a certain significant time-dependency was only obtained for the amount of vWF-stained pixels. It was found that in the x25 magnification, vWF-stained pixels were higher for both 2 and 4 meshes groups following incubation of 2 minutes compared to 5 and 10 minutes (Fig 4). This can be attributed to the ADAMTS-13 activity which lasted longer and disassembled the vWF strings to smaller fragments and monomers that were disconnected from the cell surface and therefore were not counted in the analysis.

The current woven-wire mesh design is an improvement version of our "first generation" weight design, previously reported by Rotman et al [4]. There, a constant pressure upon the cells was applied using pre-cut pieces of commercially available short peripheral catheter. That way, only one magnitude of pressure could be examined and more importantly, shear stress events could not be isolated (since we wish to only examine static mechanical pressure which is dominating in IV patients) due to spontaneous uncontrolled movements of the catheters weights upon the cell monolayer. Here, each mesh was designed to precisely accommodate each well (within the 12-well plate), therefore it cannot move parallel to the monolayer and unwanted shear forces are eliminated. Moreover, the option to apply several combinations of meshes (1–4 meshes) which correspond to elevated pressure values, imparts modularity to the system and allow us to span almost the entire (non- uniform) pressure scale which was previously shown to be exerted upon the vein wall [3]. It is important to clarify that the calculation of the contact area using the flat ellipse-shaped contact surface as described in details in S1 File was only aimed for an approximation of the exerted pressure, and the actual pressure is underestimated because of the non-flat shape of the apical side of the ECs. However, as the study purpose was to examine the ECs response to different gradual magnitudes of exerted pressures, the absolute magnitude of pressure is actually irrelevant but only the comparative response of the different treatment groups.

The main goal was to provide evidence that ECs are pressure-sensitive rather than examining a quantitative response to a specific pressure value.

In this context It should be further clarified that the mesh weights were not designed to exert compression on all the cells in the monolayer (but only upon cells underlying the ellipsed-shaped contact regions (S1 File, S2 Fig)), as not all ECs which lining the vein wall *in vivo* "feel" the pressure of the SPC. However, since adjacent cells in a monolayer develop communicative cell-cell interactions through adherens junctional zones, then cells which were mechanically compressed under the mesh and consequently activated, stimulated their neighbors to undergo the similar process. The mesh porosity structure further enables free passage for gas exchange between cells and environment.

It was found that the ELISA specification parameters were not sensitive enough to detect changes in the secreted amounts when the pressure increments from one treatment group to another was on the order of one mesh (68 Pa). Therefore, treatment groups were chosen to be 2 and 4 meshes. These groups were further consistent with the pressures values (136-272Pa) which are mainly experienced by the vein wall while the catheter *in situ* [3].

The results of the Apoptosis test ruled out the possibility of cell disruption to be the source for the secreted vWF and IL-8, and verified that the source for the enhanced secretion of both vWF and IL-8 is indeed the regulated release of WPB (was reveled through the experiments with the H_2_O_2_ as a negative control) which was triggered by the stress state induced- weights mechanical compression. The apoptosis analysis was also supported by cell morphology examination which remained intact following exposure to the mechanical pressure.

It can be therefore assumed, with high level of certainty, that the ECs feel the mechanical stress of the mesh weights, "turn on" a stress mode and activate inflammatory reaction which involves, inter alia, the secretion of vWF and IL-8. Cases of larger mechanical pressure (by applying 6–8 meshes) caused a massive destruction of the cells monolayer and very high rates of apoptosis and necrosis as well as low viability of the cultured cells. Since our aim was to evaluate the secretion of both biomarkers from living cells reacting to a stress-induced situation we examine the cells reaction under up to 4 units of mesh which as was mentioned above did not cause cells death and yielded a controlled release of both biomarkers.

Shear stress, cyclic stress and hydrostatic pressure were all been thoroughly investigated regarding their inflammatory impact on vascular ECs [24–26]. It was further shown that fluid shear stress increase vWF secretion (within few hours) from cultured endothelial cells when compared to static conditions [27]. The present study describes, for the first time, the dose-dependent modulation of vWF and IL-8 induced by mechanical compression of a foreign extrinsic body. Changes of compression forces may be developed in catheterized peripheral veins where the vein and the catheter come into contact, while *in situ* [3, 4](Coronary Stents also produce this kind of pressure, however of much larger scale that have been shown to be lethal for ECs [28]). Recently, we have demonstrated using a computational simulation an Intravenous procedure in which a short peripheral catheter is advanced into a realistic model (geometry & properties) of peripheral vein, that the catheter applies contact mechanical pressure of about 50 to 3000 Pa on the inner endothelial layer of the vein[3]. Considering the results of the present study, this pressure most likely enhances inflammatory processes within the ECs by secretion of the vWF and the IL-8 biomarkers. The above reported findings consolidate our hypothesis and constitute additional validation as to the role of contact pressure in the development of SPCT. We can confidently assume that the previous simulation- obtained stresses acting on the vein wall have an influential role in mediating the inflammation/thrombophlebitis often occurred in IV patients. Consequently, it can be speculated that minimizing or even eliminating the contact between the SPC and the vein wall will mitigate the pressure acting on the ECs layer thereby reducing the secretion of inflammatory biomarkers which in-turn will lessen the incidence of SPCT.

In conclusion, as SPCs are ubiquitous in today’s healthcare environment, yet complications related to their use, particularly SPCT, are of high incidence, the importance of delving in its pathogenesis is a fortiori invaluable. The present study confirmed that ECs are sensitive to mechanically-induced pressure and initiate inflammatory reaction in response; as greater is the exerted load so is the secretion of the inflammatory biomarkers. New biomechanically-oriented strategies concerning the contact pressure exerted by the SPC may enhance our understanding of the etiology of SPCT and hopefully improve the quality of care.

## Supporting Information

S1 Fig An example (4 meshes group) for topographic color map (right panel) generated from the corresponding immunofluorescence image (left panel). The color maps images were then converted to gray level images in order to generate histogram distribution for each treatment group in each incubation time and magnification. The red color indicates high intensity pixels and the blue stands for lower ones.
(TIF)

S2 Fig Scanning electron microscopy image of the mesh including the ellipses and rectangle drawings used to calculate the effective contact surface between the mesh and the culture dish. Scale bar length is 1mm.
(TIF)

S3 Fig Flow cytometric analysis of the apoptosis/necrosis test- Annexin V/PI assay (FL1-H- Annexin V channel, FL2-H- PI channel). HUVEC were treated with zero to four units of mesh or pretreated with 100μL H_2_O_2_, for 24,48,72,96 hours. The diagrams show representative distributions for the 96 hours incubation time of one out of three conducted experiments. As clearly seen for all the treatment groups, more than 90% of the cell population was found viable (both Annexin V and PI negative, quadrant 1- Q_1_).
(TIF)

S1 File Calculation of the pressure exerted by one mesh weight. (DOCX)

S2 File FACS and Alamar Blue viability tests. (DOCX)
