# Supplementary material for: Mechanical Compression Effects on the Secretion of vWF and IL-8 by Cultured Human Vein Endothelium
Source: PLoS One. 2017 Jan 12;12(1):e0169752. doi: 10.1371/journal.pone.0169752 (PMC5230793; doi:10.1371/journal.pone.0169752)
Supplement: S1 File — (DOCX) [file pone.0169752.s004.docx]

**S1 File. Calculation of the pressure exerted by one mesh weight.**

The net force acting on the cultured endothelial layer is the sum of the buoyancy force (according to Archimedes principle) exerted by the cultured medium and the mesh's weight, and can be described by the following equation:

$\overrightarrow{F}=m\overrightarrow{g}-\rho V\overrightarrow{g}$ (1)

Where $m$ equals to a one mesh mass, $\rho$ to the density of the cell's medium and $V$ is the volume of the submerged mesh. $\overrightarrow{g}$ is the gravitational acceleration. One mesh's volume equals the ratio between its mass which is 0.37$\text{g}$, and its density which correspond to that of stainless-steel 316- 8$g/\text{cm}^{3}$. The total sum up to$V=\text{0.046cm}^{3}$. Assuming now that the medium density is approximately that of a water in 37˚C, that is 1$g/\text{cm}^{3}$, we can derive the net force exerted by one mesh, on the ECs monolayer- $\overrightarrow{F}=$0.00365 $Kg\cdot m/s^{2}$. For calculation of the effective contact surface area between the mesh and the culture dish we utilized scanning electron microscopy (SEM). As can be seen in S2 Fig, the mesh is comprised of equal sized-ellipse shaped contact surfaces. The area of each ellipse (~0.2$mm^{2}$) was estimated by measuring the length of its major and minor axes. The total number of ellipses can be obtained by dividing the total area of the mesh surface ($\pi d^{2}/4$ where $d=21.9mm$) with the area of a rectangle which contains one whole ellipse (A total of four quarter-ellipse) which overall sums up to 314 ellipses in each mesh. Finally, by multiplying the number of ellipses within the mesh with the calculated ellipse-surface area we could derive the total effective contact surface area of the mesh with the cells culture dish. The pressure exerted by one mesh weight can be now obtained by dividing the net force with the effective surface area which equals to 68 Pa. Similarly, the pressure exerted by 2 and 4 units of mesh is 136 Pa and 272 Pa, respectively.
