# Supplementary material for: Mechanical Compression Effects on the Secretion of vWF and IL-8 by Cultured Human Vein Endothelium
Source: PLoS One. 2017 Jan 12;12(1):e0169752. doi: 10.1371/journal.pone.0169752 (PMC5230793; doi:10.1371/journal.pone.0169752)
Supplement: S2 File — (DOCX) [file pone.0169752.s005.docx]

**S2 File. FACS and Alamar Blue viability tests.**

Viability of the cells following indwelling of meshes within the culture medium (without contacting the cells) was measured using Alamar Blue™ assay (Biosource, Camarillo, CA, USA). The Alamar Blue assay measures cytotoxicity and cell proliferation via metabolic activity through the chemical reduction of Alamar Blue™ by living cells. The treated cells were incubated at 37˚C in 10% of Alamar Blue™ solution and 90% of culture medium for 24-96 hours. The medium was sampled into 96-well microplate. Then, samples fluorescence was measured using Spectrophotometer (Multiskan™ GO, Thermo) at excitation wavelength of 570nm and emission wavelength of 600nm, and normalized to the pure solution of Alamar Blue™ without the cells. The results disproved the toxicity of the mesh weights towards the ECs. It was shown that indwelling of 1 mesh for 96 hours yielded similar metabolic rate compared to control (86%±3 Vs. 89%±5). This behavior was also observed for shorter incubation times (24, 48 and 72 hours) and was not influenced by the number of meshes which submerged into the wells.

To verify that the ECs do not irreversibly squashed under the mesh weights and remain viable through the course of the experiments, Annexin V-FITC and PI staining was performed. The average values (3 experiments) for annexin V-negative/PI-negative (viable cells) following incubation for 96 hours, were 97.2, 94.7, 94.1 and 92.3% for the 4 meshes, 2 meshes, control and H_2_O_2_, respectively (S3 Fig.).
